# Financial burden of heart failure in Malaysia: A perspective from the public healthcare system

**DOI:** 10.1371/journal.pone.0288035

**Published:** 2023-07-05

**Authors:** Siew Chin Ong, Joo Zheng Low

**Affiliations:** 1 Discipline of Social and Administrative Pharmacy, School of Pharmaceutical Sciences, Universiti Sains Malaysia, Gelugor, Penang, Malaysia; 2 Hospital Sultan Ismail Petra, Ministry of Health Malaysia, Kuala Krai, Kelantan, Malaysia; The University of Mississippi Medical Center, UNITED STATES

## Abstract

**Background:**

Estimating and evaluating the economic burden of HF and its impact on the public healthcare system is necessary for devising improved treatment plans in the future. The present study aimed to determine the economic impact of HF on the public healthcare system.

**Method:**

The annual cost of HF per patient was estimated using unweighted average and inverse probability weighting (IPW). Unweight average estimated the annual cost by considering all observed cases regardless of the availability of all the cost data, while IPW calculated the cost by weighting against inverse probability. The economic burden of HF was estimated for different HF phenotypes and age categories at the population level from the public healthcare system perspective.

**Results:**

The mean (standard deviation) annual costs per patient calculated using unweighted average and IPW were USD 5,123 (USD 3,262) and USD 5,217 (USD 3,317), respectively. The cost of HF estimated using two different approaches did not differ significantly (p = 0.865). The estimated cost burden of HF in Malaysia was USD 481.9 million (range: USD 31.7 million– 1,213.2 million) per year, which accounts for 1.05% (range: 0.07%–2.66%) of total health expenditure in 2021. The cost of managing patients with heart failure with reduced ejection fraction (HF*r*EF) accounted for 61.1% of the total financial burden of HF in Malaysia. The annual cost burden increased from USD 2.8 million for patients aged 20–29 to USD 142.1 million for those aged 60–69. The cost of managing HF in patients aged 50–79 years contributed 74.1% of the total financial burden of HF in Malaysia.

**Conclusion:**

A large portion of the financial burden of HF in Malaysia is driven by inpatient costs and HF*r*EF patients. Long-term survival of HF patients leads to an increase in the prevalence of HF, inevitably increasing the financial burden of HF.

## Introduction

Heart failure (HF) results from abnormalities or damage to the heart’s structure or function, reducing cardiac output and raising intra-cardiac pressures at rest or during exertion [1]. About 64 million people worldwide have HF, with 50% having severe HF (symptomatic HF at rest) [2]. Malaysia had one of the highest HF prevalence rates in Southeast Asia, with 721 cases per 100,000 persons in 2017, an increase of 7.7% from 669 cases per 100,000 persons in 1990 [3].

HF requires frequent hospitalisation and reduces life expectancy and quality of life. HF’s 30-day readmission rate ranged from 6.3% to 56% [4, 5]. Six-month all-cause readmission was 23% to 50% [6–8]. In Malaysia, 13% to 18.1% of HF patients were readmitted within 30 days [9, 10]. Furthermore, the readmission rate of HF increased steadily from 16.6% in 2007 to 19.6% in 2015 [10].

Management of HF is associated with increased healthcare resource utilisation and cost, leading to an increase in economic and clinical burden during the acute decompensated state and for a prolonged period after that [11]. HF consumes about 1–2% of developed countries’ annual healthcare budgets [8]. Cook et al. estimated the cost burden of HF across 197 countries using published estimates of HF economic burden [12]. The direct cost of HF consumed 1.1% and 0.11% of total health expenditure in high and middle-low-income countries, respectively. However, recently published data from Korea [13] and the Philippines [14] showed that the direct cost of HF was higher than the value estimated by Cook et al. These changes could be due to the increase in the countries’ gross domestic product (GDP) [15], and the direct cost of HF is positively correlated with the GDP [12, 16]. In addition, the risk of developing HF increases with age, so HF prevalence and costs are expected to rise with an ageing population [13, 17, 18]. Furthermore, different HF phenotypes categorised using left ventricular ejection fraction (LVEF) accrued cost differently [5, 19–21]. In Malaysia, 66.7% of HF patients were classified as LVEF <40% [9]–which was higher than in other European (59.0%) [22] and East Asia countries (21.0–35.2%) [23, 24]. Considering the increasing prevalence of HF, growing GDP, and different HF phenotypes distribution in Malaysia, the annual economic burden of HF is estimated to be higher than the value reported by Cook et al. for Malaysia (i.e., USD 12 million).

The previous cost analysis study focused on estimating the mean annual cost of HF by cost components for different HF phenotypes (categorised by LVEF), disease severities (categorised by New York Heart Association functional classification [NYHA]), and underlying comorbidities [25]. Subsequently, the main focus of the current study was to estimate the population-level economic burden of HF in Malaysia. Furthermore, the present study is a continuous work from the previous study by comparing the costs estimated using the unweighted average and inverse probability weighting (IPW) methods to provide insight into the impact of censored data on the average costs.

One approach to justify allocating healthcare resources to a specific condition would be to provide data on the extent of our healthcare system’s burdens for that condition. Thus, the present study aimed to estimate the overall economic burden of HF from the perspective of the public healthcare system. Identifying the HF phenotypes with the highest cost burden will help prioritise the scarce healthcare resources.

## Methodology

### Measurement of the direct cost of HF

The methodology for estimating the direct cost of HF has been described in detail in the previous cost analysis study [25]. The study obtained ethical approval from the Ministry of Health Medical Research and Ethics Committee (NMRR-20-2379-56411(IIR)). Since all patient-related information in the database is anonymous, it is not necessary to obtain the patients’ informed consent. Briefly, adult HF patients aged ≥ 18 who sought medical treatment in tertiary hospitals (Penang General Hospital, Serdang Hospital and Hospital Queen Elizabeth II) were included in the study. The patients were categorised into three groups, namely: HF with reduced ejection fraction (HF*r*EF) (LVEF ≤40%), HF with mildly-reduced ejection fraction (HF*mr*EF) (LVEF 41–49%), and HF with preserved ejection fraction (HF*p*EF) (LVEF≥50%). An annual prevalence-based cost-of-illness approach was adopted from Malaysia’s Ministry of Health (MoH) perspective. A bottom-up approach was used, whereby data on the quantity of healthcare resource utilisation were retrospectively extracted from patients’ medical records. The total annual direct cost per patient from each HF phenotype was calculated by adding all cost components from outpatient and inpatient settings. Five cost components were identified: outpatient visit, hospitalisation, medication, diagnostic, and procedure. The cost of each component was generated by the product of the respective utilisation frequency and their associated unit cost. The costs were presented in Ringgit Malaysia (RM) and US dollars (USD). The conversion rate was based on the 2021 purchase power parity (PPP) (USD 1 = RM 1.59) [26].

### Analysis

Unweighted average and IPW were used to estimate annual HF cost per patient. For the unweighted average, the cost incurred in the outpatient setting was first divided by the duration of follow-up (in months) for each patient, then converted to annual cost by multiplying by 12 months and dividing by the total number of patients to obtain the mean annual outpatient cost per patient. The mean annual inpatient cost per patient only includes hospitalised patients.

This study employed broad inclusion criteria to achieve a sufficient sample size. However, not all the patients had complete cost data for one year of follow-up (censored patients). An IPW estimator proposed by Bang and Tsiatis [27] was applied to estimate the total healthcare cost of HF with censored data. The equation used to calculate the mean annual cost of HF using the IPW method is shown below:

$$Mean\;annual\;cost=\frac{1}{n}\left[\;\sum_{i}^{n}\frac{\Delta_{i}A_{i}\left(t_{i}\right)}{S_{c}(t_{i})}\;\right]$$ (1)

*n* = Total sample size, including censored and uncensored patients

*t*_*i*_ = Time to fix endpoint, death, or loss to follow up for each patient (in months)

*A*_*i*_ *(t*_*i*_*)* = The cumulative cost until the time, *t* for a patient, *i*

*S*_*c*_ *(t*_*i*_*)* = The probability of being uncensored beyond time, *t*

The costs incurred during each interval were the sum of all cost components during that interval for all censored and uncensored patients. The interval cost was then weighted by the inverse probability of being uncensored, *S*_*c*_*(t*_*i*_*)*, to account for censored data. *S*_*c*_*(t*_*i*_*)* was derived using the Kaplan-Meier method for censoring [27]. The mean annual cost of HF was calculated by adding all weighted interval costs and dividing by the number of eligible patients. The cost derived using the IPW estimator was used as the base case estimation of the mean total healthcare cost of HF.

The continuous variables were reported as mean (standard deviation, SD) or median (interquartile range, IQR) based on the distribution of data. The categorical variables were presented as numbers and percentages. The annual total healthcare costs of HF were presented as the mean and standard deviation, and these were used to compare the mean difference of costs across different LVEF using parametric tests such as analysis of variance (ANOVA). Student’s t-test was used to compare the cost estimated using unweighted average and IPW. The assumption made by using parametric comparative analysis was that the sample size was large enough to comply with the central limit theorem. Despite right-skewed cost data, the estimates were reported in mean so they could be used for economic evaluation to guide decision-making [28, 29]. Statistical significance was set at the value of p < 0.05. All analysis was conducted using Microsoft Excel^®^ (Microsoft, USA) and R [30].

### Estimation of economic burden of heart failure

The population-level economic burden of HF in Malaysia was estimated for the adult population from the perspective of the public healthcare system. The population-level economic burden of HF was estimated based on LVEF and age categories. The economic burden of HF was calculated by multiplying the mean annual cost of HF derived from the cost analysis [25] by the population of chronic HF (categorised by LVEF and age categories). The mean annual costs of HF were stratified according to LVEF and age categories.

The population of HF in Malaysia was calculated by applying the prevalence of HF in Malaysia to the Malaysian adult population. The Department of Statistics Malaysia estimated Malaysia’s population for 2021 was 32.66 million [31], with 72% aged at least 18 years [32]. The Global Burden of Disease, Injuries, and Risk Factors (GBD) Study estimated 720.6 HF patients per 100,000 population [95% confidence interval (CI) 624.6–820.1 HF patients per 100,000 population] in 2017 [3]. The adult HF population was subsequently estimated to be 169,474 (95% CI 146,896–192,875). The diagnosis rate was estimated based on expert opinion, and the proportion of prescriptions filled in the public sector [33] was used to determine the base case estimation of HF population that was followed up in public institutions. The base case estimate of HF population was then categorised according to LVEF using the prevalence of HF*p*EF, HF*mr*EF, and HF*r*EF as derived from the Malaysia Heart Failure Registry (MyHF) [9]. Additionally, the breakdown of HF patients by different age groups was calculated based on values obtained from a local study that evaluated the readmission rate of HF-related hospitalisation in patients aged ≥ 20 years [10]. Since the youngest patient in the cost analysis study was 24 years old, the prevalence of the Malaysian population aged ≥ 20 years (68.4%) was used to estimate the total number of HF patients categorised with different age groups.

### Sensitivity analysis

Two one-way sensitivity analyses were performed to account for the uncertainties in estimating HF’s healthcare cost and prevalence. In the first one-way sensitivity analysis, the direct cost of HF estimated using the IPW method varied between the 95% CI but the HF patient numbers across different phenotypes and age groups were kept constant. In the second one-way sensitivity analysis, the estimated number of HF with different phenotypes and age groups were varied, but the direct cost of HF remained constant.

A two-way sensitivity analysis evaluated the combined effects of varying the cost components and the number of HF patients with different phenotypes and age groups. The two-way sensitivity analysis evaluated the best-case and worst-case scenarios using the lower and upper limits of the cost components and the number of HF patients.

## Results

### Sociodemographic characteristics

A total of 329 patients were included in the study. Most participants were males (n = 270, 82.1%), and the mean age (SD) was 54.6 (11.7) years (S1 Table, see electronic supplementary material [ESM]). Hypertension (68.4%) was the most prevalent comorbidity, followed by coronary artery disease (56.2%), dyslipidaemia (47.1%), and type II diabetes mellitus (DM) (43.5%). Only 30.7 of the study population had at least one hospitalisation due to HF (hHF) during the follow-up period.

### Direct cost

**Fig 1:**
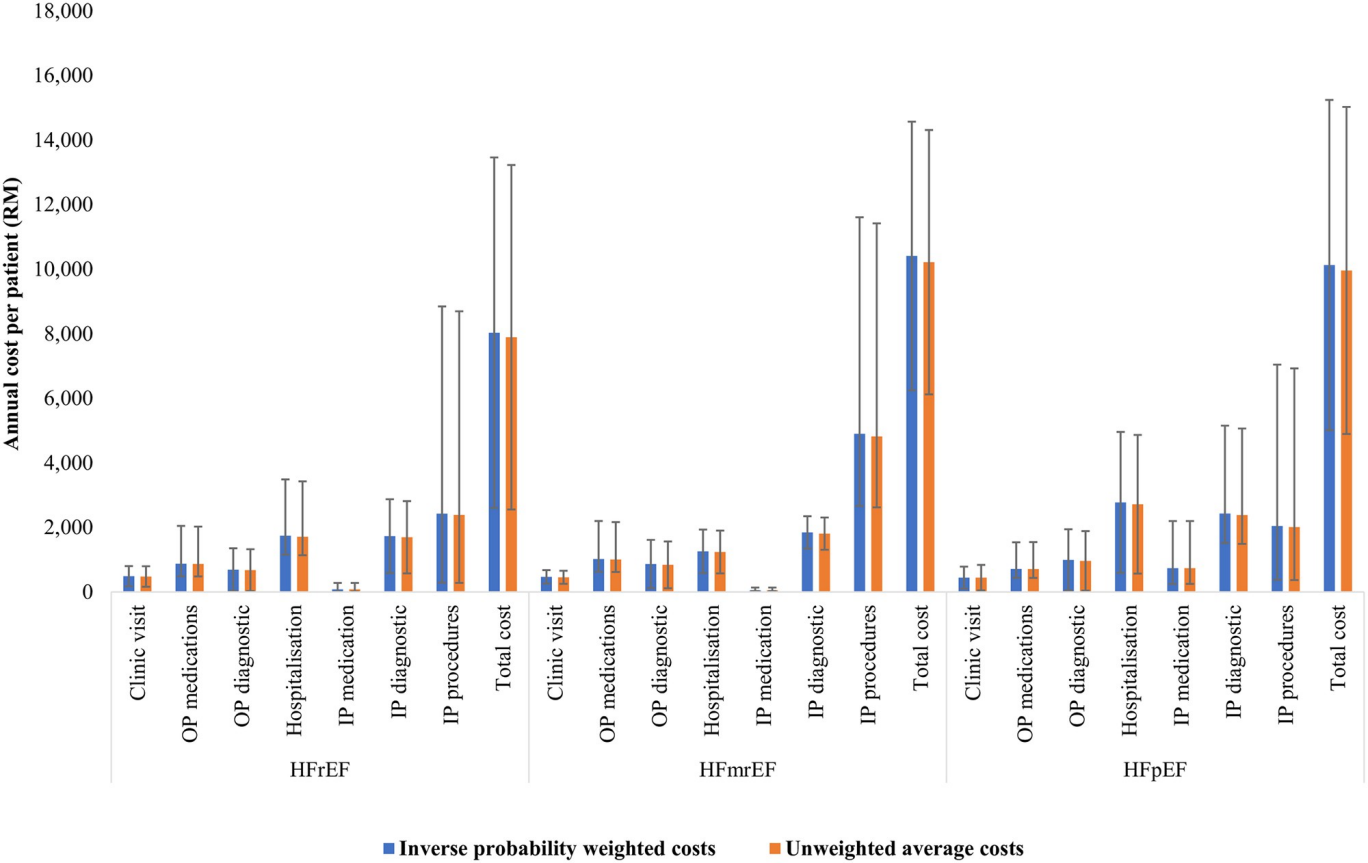
Annual cost per patient (in RM). HF*m*rEF: heart failure with mildly reduced ejection fraction; HF*p*EF: heart failure with preserved ejection fraction; HF*r*EF: heart failure with reduced ejection fraction; IP: inpatient; OP: outpatient; RM: ringgit Malaysia. Inverse probability weighted costs were estimated using Bang & Tsiatic estimator [27]. Unweighted average costs were estimated by dividing the total cost by the total months of follow-up and transformed to annual cost by multiplication of 12 months.

The mean (SD) annual cost per patient estimated using the IPW method was RM 8,295 (RM 5,274) [USD 5,217 (USD 3,317)], with inpatient costs accounting for 74.8% of the total costs (Fig 1). HF*mr*EF encountered the highest costs [RM 10,410 (RM 4,162), USD 6,547 (USD 2,618)], followed by HF*p*EF [RM 10,128 (RM 5,117), USD 6,368 (USD 3,218)] and HF*r*EF [RM 8,033 (RM 5,430), USD 5,052 (USD 3,415)]. The differences in the mean annual cost across HF phenotypes were not statistically significant (*p =* 0.778).

The mean annual cost per patient calculated using the unweighted average was RM 8,146 (RM 5,187) [USD 5,123 (USD 3,262)]. S2 Table (see ESM) summarises the mean difference of annual HF cost per patient estimated using the unweighted average and IPW method. The mean total annual costs estimated by the IPW method were higher than the unweighted average but not statistically different [mean difference (MD) = RM 148, 95% CI: -RM 870–731 (MD = USD 93, 95% CI: -USD 547–460), p = 0.865]. The annual costs estimated using both methods were not statistically different across HF phenotypes and cost components, p>0.05.

### Economic burden of heart failure

Cardiologists from tertiary hospitals estimated that the overall diagnosis rate of HF in Malaysia was 80%. Based on the proportion of medicine use in public and private sectors in 2016, 64.5% of the diagnosed HF patients were followed up in public healthcare institutions [33]. Therefore, 87,448 HF patients (95% CI 75,798–99,523) received HF treatment in public healthcare institutions. According to MyHF, the prevalence of HF*p*EF, HF*mr*EF, and HF*r*EF were 21.8%, 11.5%, and 66.7%, respectively [9]. Thus, the total number of HF*p*EF, HF*mr*EF, and HF*r*EF patients who sought medical treatment in the public healthcare system was 19,064 (95% CI 16,524–21,696), 10,057 (95% CI 8,717–11,445) and 58,328 (95% CI 50,558–66,382), respectively.

The base case estimated annual direct cost of HF management from the public healthcare system’s perspective in Malaysia was RM 766.3 million (USD 481.9 million) (Table 1). Managing HF*r*EF patients accounted for 61% of the total financial burden of HF in Malaysia despite having the lowest mean annual cost. The high disease burden of HF*r*EF drove the main economic burden of HF in Malaysia.

**Table 1:** Base-case estimated number of patients categorised in different phenotypes and age groups and their base-case cost of management.

	Number of patients	Annual cost per patient (mean cost) (RM)	Estimated cost per annum (RM million)
**HF phenotypes** ^†^			
HF*p*EF	19,064	10,128	193.0
HF*mr*EF	10,057	10,410	104.7
HF*r*EF	58,328	8,033	468.5
Total	87,448	28,568	766.3
**Age groups** ^‡^			
20–29	1,110	4,086	4.5
30–39	3,243	6,010	19.5
40–49	8,190	9,279	76.0
50–59	18,188	7,368	134.0
60–69	24,740	9,136	226.0
70–79	18,914	8,204	155.2
80+	8,668	9,271	80.4

HF: heart failure; HF*m*rEF: heart failure with mildly reduced ejection fraction; HF*p*EF: heart failure with preserved ejection fraction; HF*r*EF: heart failure with reduced ejection fraction; RM: ringgit Malaysia

^†^ The proportion of the population aged ≥18 years was used to estimate the HF population.

^‡^ The proportion of the population aged ≥20 years was used to estimate the HF population because the youngest HF patient in this cohort was 24 years.

The annual cost of HF per patient increased with age, from RM 4,086 (USD 2,570) for the age group 20–29 to RM 9,271 (USD 5,831) for the age group 80+ years. Although the mean annual costs of HF per patient were not significantly different between patients with ages <60 years and ≥ 60 years [MD = RM 997, 95% CI: -RM 1,053–1,275, (MD = USD 627, 95% CI: -USD 666–802, p = 0.851)], the overall cost of HF increased by 68.7% to RM 226.0 million (USD 142.1 million) for age group 60–69 years as compared to 50–59 years due to growing number of patients living with HF. Conversely, the financial burden of managing HF decreased to RM 80.4 million (USD 50.6 million) for the age group of 80+ years. The cost of managing HF in patients from 50–79 years accounted for 74.1% of the total financial burden of HF in Malaysia.

Varying the direct cost of managing HF (first one-way sensitivity analysis) has a more significant impact on the overall financial burden of HF than changing the number of patients (Table 2). In the first sensitivity analysis, the direct cost of managing HF was RM 58.2 million– 1,695 million (USD 36.6 million– 1,066 million) compared to RM 664.2 million– 872.1 million (USD 417.7 million– 548.4 million) in the second one-way sensitivity analysis. Sensitivity analysis confirmed that HF*r*EF has the highest economic burden. The two-way sensitivity analysis considered the best- and worst-case scenarios showed that the financial burden of HF cost between RM 50.5 million– 1,929 .0 million (USD 31.7 million– 1,213.2 million). Thus, the HF financial burden was estimated to account for 1.05% (range: 0.07%–2.66%) of Malaysia’s total health expenditure in 2021 (S3 Table, see ESM) [34].

**Table 2:** One- and two-way sensitivity analyses for the estimated annual direct cost of HF in each phenotype and age groups.

	Range of number estimated of patients	Range of estimated direct cost (RM)	First one-way sensitivity analysis ^†^ (RM million)	Second one-way sensitivity analysis ^‡^ (RM million)	Two-way sensitivity analysis (best case scenario) (RM million)	Two-way sensitivity analysis (worst case scenario) (RM million)
**HF phenotypes**						
HF*p*EF	16,524–21,696	719–19,159	13.7–365.2	167.3–219.7	11.9	415.7
HF*mr*EF	8,717–11,445	734–18,138	7.4–182.4	90.7–119.1	6.4	207.6
HF*r*EF	50,558–66,382	637–19,671	37.1–1147.4	406.1–533.2	32.2	1,305.8
Total	75,798–99,523	2,089–56,967	58.2–1695.0	664.2–872.1	50.5	1,929.0
**Age groups**						
20–29	962–1,263	679–6,374	0.8–7.1	3.9–5.2	0.7	8.1
30–39	2,811–3,690	677–9,482	2.2–30.7	16.9–22.2	1.9	35.0
40–49	7,099–9,321	736–26,841	6.0–219.8	65.9–86.5	5.2	250.2
50–59	15,765–20,700	678–17,951	12.3–326.5	116.2–152.5	10.7	371.6
60–69	21,444–28,157	574–19,617	14.2–485.3	195.9–257.2	12.3	552.3
70–79	16,394–21,525	668–17,018	12.6–321.9	134.5–176.6	10.9	366.3
80+	7,513–9,865	987–9,653	8.6–83.7	69.7–91.5	7.4	95.2

HF: heart failure; HF*m*rEF: heart failure with mildly reduced ejection fraction; HF*p*EF: heart failure with preserved ejection fraction; HF*r*EF: heart failure with reduced ejection fraction; RM: ringgit Malaysia

^†^ One-way sensitivity analysis by varying the direct cost of HF but the HF patient numbers across different phenotypes but age groups was kept constant.

^‡^ One-way sensitivity analysis by varying the estimated number of HF patients with different phenotypes and age groups but the direct cost of HF was kept constant.

## Discussions

The estimated mean annual healthcare cost per HF patient in Malaysia was RM 8,295 (RM 5,274) [USD 5,217 (USD 3,317)], with inpatient costs contributing about three-quarters (74.8%) of the total costs. This translated to a significant financial burden on the public healthcare system, especially for patients with HF*r*EF and aged between 50 and 79 years, which accounted for 61% and 74.1% of the total cost burden of HF in Malaysia, respectively.

This study employed broad inclusion criteria to achieve a sufficient sample size. However, the main drawback of such an approach was that not all the patients had complete cost data for one year of follow-up. Considering all observed or uncensored cases only (ignoring censored costs) will provide an inaccurate estimate of the actual mean total cost [35, 36]. An IPW estimator was applied to accurately estimate the total healthcare cost of HF [27]. The IPW estimator was chosen over other techniques that could handle the time-restricted mean cost because the former used single-record-per-subjects survival data. Furthermore, the IPW estimator with known cost histories provides robust mean and standard error estimates across different censoring mechanisms [37] and with mild to moderate degrees of data censoring (<18%) [38]. The periods with data censored observed in this study were 1.44% (S4 Table, see ESM). Thus IPW estimator is a reasonable and appropriate approach to handling censored cost data [39].

There was no significant difference in the mean annual cost of HF estimated using the unweighted average and IPW method in the current study. This might be due to the current study duration not being long enough to observe a significant difference in the cost. Another study that employed a similar method and duration of follow-up also found that the mean total annual cost of HF estimated by the unweighted average and IPW method showed no significant difference [40]. However, another study which estimates the cost of oral cancer patients with a longer duration of follow-up (4–5 years) observed a significant difference in the mean cost estimated using the unweighted average and IPW estimator [41]. The probability of having censored costs being low with a short duration of follow-up could cause the low impact of censored costs on estimating the actual mean total cost in the current study [42].

A systematic review that included cost studies from North America, South America, Europe, Asia and Africa reported that the annual healthcare cost related to HF per patient was between USD 1,096 and USD 101,886 in 2021 [43]. Another systematic review by Lesyuk et al. that included the USA, Europe, Africa and Asian countries found that inpatient admission costs accounted for about 44% to 96% of the overall direct cost of HF [44]. The wide variation in the costs reported across different countries may be mainly attributed to the relative cost variation of healthcare resources utilised to treat HF, treatment protocol, and, most importantly, the methodology used in calculating the costs. Additionally, the differences could also be explained by the vast discrepancy in resource allocation by each country for their healthcare system in managing HF [12].

From the public healthcare perspective, the direct cost of managing HF in Malaysia was RM 766.3 million (range: RM 50.5 million– 1,929 .0 million) (USD 481.9 million, range: USD 32.0 million– 1,213.2 million), which account for 1.05% (range: 0.07%–2.66%) of Malaysia’s total health expenditure in 2021. The global economic burden of HF was USD 108 billion per year in 2012 [12]. Developed countries devoted 1–2% of their total health expenditure to managing HF [8]. On average, the direct cost of HF consumed 1.1% and 0.11% of total health expenditure in high and middle-low-income countries in 2012, respectively [12]. The annual economic burden of HF in developed countries, such as the USA (USD 42.1 billion) [45], Korea (USD 625.6 million) [13] and the UK (USD 3 billion) [46], was much higher than in Malaysia. Healthcare expenditure is positively correlated with GDP [12, 16]. Thus, developed countries can allocate more resources to the healthcare sector. Conversely, developed countries like Ireland reported a lower economic burden (USD 243.9 million) [47] than Malaysia because the Irish population is significantly lower than Malaysia [48] and hence lesser people living with HF. Although Brazil has a higher GDP than Malaysia [49], the HF economic burden of Brazil (USD 420.3 million) [50] was lower than Malaysia because the study was conducted two decades ago. Advancement in the treatment of HF, including device therapy and newer pharmacology therapy, which are more expensive, has increased the healthcare cost of managing HF. Even though the GDP of Malaysia and the Philippines is similar, the economic burden of the Philippines was lower (USD 52.6 million) because only hospitalisation cost was considered when estimating the economic burden [14].

The direct cost burden of Malaysia reported by Cook et al. was USD 12 million in 2012 or 0.11% of total health expenditure, much lower than the cost burden estimated in this study. The discrepancy was caused by the economic burden of HF in countries without published data was extrapolated by Cook et al. using data from countries with published estimates, such as the USA and Brazil. In addition, the increase in the economic burden of HF in Malaysia was mainly attributed to the steady rise in the prevalence of HF [3].

The current study estimated the economic burden of HF in Malaysia using cost data calculated from the bottom-up approach [25]. Unlike other studies in which the costs were derived from insurance claims [13, 45] and hospital casemix database [50], the bottom-up approach can segregate the costs according to disease severities and other categories because detailed data was collected from each patient [51]. The current study reports the economic burden by HF phenotypes (categories using LVEF) and age groups, which enables the decision-makers to identify the main cost components and hence design policies to curb the huge economic burden of HF in Malaysia. The costs estimated from the casemix database were attributed to the primary diagnosis, and segregation of costs would be difficult [51].

We observed that the cost burden of managing HF*r*EF patients was the highest among the three HF phenotypes because the prevalence of HF*r*EF was the highest among the HF population in Malaysia [9] and Asia [52]. HF*r*EF was more commonly found among younger patients [17, 53] and having coronary artery disease (CAD) as the aetiology of HF [53, 54]. The mean age of this study population was 54.6 years, consistent with the population of HF-related studies previously conducted in Malaysia [9, 40, 55]. The Japanese HF cohort found that the mean age of HF*p*EF was significantly older than the other two phenotypes and was the most prevalent HF phenotype (61.9%) among the study population [24]. Besides, the prevalence of the traditional risk factors for CAD, such as diabetes, hypertension, and dyslipidaemia, has increased steadily among Malaysia’s population since 2011 [56], translated into a higher risk of developing myocardial infarction and eventually leading to HF*r*EF [17, 53].

In our study, we observed that the financial burden of HF increased with age until age 69 years because the prevalence of HF increases with age [13, 17, 18]. In addition, advancing age further increases the existing risk of traditional risk factors for developing HF [17]. In addition, the cost burden of HF peaked at the age group 60–69 years because HF patients in Malaysia were diagnosed at a much younger age (approximately 60 years) [9, 40, 55]compared to patients from other Asia countries (range: 66–81 years) [5, 57, 58] and western countries (range: 71–77 years) [21, 59, 60]. Furthermore, the cost of HF was the highest during the initial HF diagnosis [21]. Besides, the prevalence of HF decreased after 69 years in Malaysia, leading to a decrease in the cost burden of HF. This observation could be due to the life expectancy of patients with HF being 5–10 years after diagnosis [61, 62]. Secondly, the mean annual cost of HF in geriatric patients (80+ years) was lower than those of younger patients because older patients were treated with more conservative strategies [58] or were unable to seek medical treatment in the hospital due to their frail condition [40].

The growing economic burden of HF can substantially affect the clinical decisions and management strategies of healthcare providers and physicians. The rising economic burden of HF may reduce the availability of specialised clinics, multidisciplinary care teams, or equipment critical to managing HF patients. N-terminal pro-brain natriuretic peptide (NT-proBNP) is a biomarker for diagnosing HF and monitoring disease progression. However, this testing service is not widely available in Malaysia due to its impact on the financial budget. In addition, the high economic burden of HF can influence the selection of treatment options. Even though more expensive therapies may be more effective, clinicians may prioritise less expensive ones due to cost considerations [63]. Besides, the economic factor also affects the treatment compliance of HF patients. Patients may have trouble accessing their medications if the cost of their prescriptions is high or their insurance does not cover them [64]. This may result in non-adherence, underdosing, or medication discontinuation, all of which can negatively impact clinical outcomes [65].

This study has the potential risk of underestimating HF’s economic burden in Malaysia. First, the economic burden was calculated from the perspective of the public healthcare system only, and the indirect cost was not considered. The direct cost associated with private healthcare institution follow-up was not considered as funding mainly sourced from patients’ out of pockets expenses or private insurance companies. However, MoH Malaysia remains the most prominent financing source for healthcare in Malaysia (i.e., 52.5% of the total health expenditure) [66]. Future studies on the economic burden of Malaysia could include the healthcare cost from the private setting because one-third of the Malaysian population received treatment in the private setting [33]. In addition, future studies can be conducted from the societal perspective to include the indirect cost associated with absenteeism and productivity loss. Secondly, the prevalence of HF in Malaysia used in this study was the estimated value for 2017, but the prevalence of HF in 2021 could be higher due to the high prevalence of risk factors for HF and longer life expectancy in patients with comorbidities [67]. Nevertheless, sensitivity analyses have been performed to address these uncertainties around HF’s economic burden. Thirdly, this study had inherent limitations associated with retrospective data collection from patients’ medical records. However, data collectors were trained to find all HF-relevant details from patients’ medical records to increase the estimation accuracy. The strength of this study was that the direct cost of HF was calculated from the resources utilised in managing HF patients in three tertiary centres using the standard cost-of-illness method and local epidemiological data to estimate the HF’s economic burden. To the best of our knowledge, the present study is the first to estimate the financial burden of HF in Malaysia nationally. The current study’s findings could help stakeholders allocate resources for funding prevention programmes by identifying risk factors for HF and early diagnosis and treatment of HF.

## Conclusion

The present study showed that HF posed a substantial financial burden to the public healthcare system. The cost of HF estimated using weighted and unweighted approaches did not differ significantly in the current study. The higher prevalence of HF*r*EF in Malaysia and the inpatient costs are the main financial burden of HF. Demographic shifts in Malaysian society will inevitably increase the prevalence of HF, which would further increase the financial burden of HF. Thus, interventions that reduce hHF and target HF risk factors to prevent new HF may have economic benefits.

## Supporting information

S1 Table Baseline demographic characteristics of heart failure patients. (PDF)

S2 Table Mean difference of cost components estimated using unweighted average and inverse probability weighting method. (PDF)

S3 Table The heart failure direct cost burden as a percentage of total healthcare expenditure in 2021. (PDF)

S4 Table Summary of censored data. (PDF)
