# Supplementary material for: Financial burden of heart failure in Malaysia: A perspective from the public healthcare system
Source: PLoS One. 2023 Jul 5;18(7):e0288035. doi: 10.1371/journal.pone.0288035 (PMC10321615; doi:10.1371/journal.pone.0288035)
Supplement: S1 Table — (PDF) [file pone.0288035.s001.pdf]

Table S1: Baseline demographic characteristics of heart failure patients.

**Patient characteristics**	**Patients, (n=329)**
Gender, n (%)	
Male	270 (82.1)
Female	59 (17.9)
Age (years), mean (SD)	54.6 (11.7)
Ethnicity, n (%)	
Malay	105 (31.9)
Chinese	98 (29.8)
Indian	50 (15.2)
Others	76 (23.1)
Duration of Heart Failure Diagnosis, n (%)	
≤1 year	235 (71.4)
2-4 years	68 (20.7)
≥5year years	26 (7.9)
Comorbidities, n (%)	
Coronary artery disease	185 (56.2)
Hypertension	225 (68.4)
Type II diabetes mellitus	143 (43.5)
Dyslipidaemia	155 (47.1)
Chronic kidney disease	44 (13.4)
Atrial fibrillation	46 (14.0)
Cerebrovascular accident	23 (7.0)
Chronic obstruct pulmonary disease	12 (3.7)
Anaemia	7 (2.1)
Others	102 (31.0)
*eGFR (mL/min/1.73m^2), median (quartiles)	38.0 (22.5, 47.1)
Baseline EF, n (%)	
HFrEF (≤40%)	263 (80.0)
HFmrEF (41-49%)	34 (10.3)
HFpEF (≥50%)	32 (9.7)
Smoker status, n (%)	
Current smoker	62 (18.8)

Ex-smoker	128 (38.9)
Not smoking	93 (28.3)
Unknown	46 (14.0)

EF: ejection fraction; eGFR: estimated glomerular filtration rate; HF*m*rEF: heart failure with mildly reduced ejection fraction; HF*p*EF: heart failure with preserved ejection fraction; HF*r*EF: heart failure with reduced ejection fraction; IQR: interquartile range; SD: standard deviation
*eGFR only available for patients with chronic kidney disease.
[#]Other ethnic included: Bajau, Dusun, Kadazan, Melanau, Murut and Iban
