# Supplementary material for: Financial burden of heart failure in Malaysia: A perspective from the public healthcare system
Source: PLoS One. 2023 Jul 5;18(7):e0288035. doi: 10.1371/journal.pone.0288035 (PMC10321615; doi:10.1371/journal.pone.0288035)
Supplement: S2 Table — (PDF) [file pone.0288035.s002.pdf]

Table S2 Mean difference of cost components estimated using unweighted average and inverse probability weighting method

	Mean difference (95% CI)			
	**Total [a]**	**HF*r*EF [b]**	**HF*mr*EF [b]**	**HF*p*EF [b]**
**Outpatient cost (RM)**				
**Clinic**	9 (-57, 38)	10 (-64, 44)	14 (-114, 86)	0.221 (-185, 185)
p-value	0.696	0.717	0.775	0.998
**Medications**	9 (-182 – 166)	8 (-208, 191)	14 (-581, 551)	1 (-413, 417)
p-value	0.928	0.935	0.958	0.993
**Diagnostic**	18 (-125, 90)	15 (-127, 97)	26 (-384, 330)	29 (-496, 438)
p-value	0.749	0.796	0.882	0.901
**Total outpatient**	35 (-256, 187)	33 (-284, 218)	56 (-728, 616)	27 (-698, 643)
p-value	0.757	0.796	0.868	0.935
**Inpatient cost (RM)**				
**Hospitalisation**	33 (-513, 447)	33 (-542, 477)	21 (-994, 953)	57 (-2,842, 2730)
p-value	0.892	0.901	0.962	0.965
**Medications**	0.105 (-113, 113)	0.095 (-58, 58)	0.001 (-118, 118)	0.332 (-1,879, 1878)
p-value	0.999	0.997	0.999	0.999
**Diagnostic**	36 (-380, 309)	34 (-368, 298)	36 (-768, 697)	46 (-3,523, 3,430)
p-value	0.840	0.838	0.913	0.977
**Procedure**	44 (-1,785, 1,697)	42 (-1,915, 1,830)	80 (-9,787, 9,626)	34 (-6,415, 6,348)
p-value	0.961	0.965	0.985	0.991

**Total inpatient**	113 (-2,182, 1,958)	109 (2,311 – 2,092)	137 (-10,381 – 10,107)	137 (-10,763, 10,491)
p-value	0.915	0.922	0.976	0.978
**Total**	148 (-870, 731)	142 (-993, 852)	193 (-2,075, 1,923)	164 (-2,598, 2,492)
p-value	0.865	0.881	0.940	0.967

CI: confidence interval; HF*m*rEF: heart failure with mildly reduced ejection fraction; HF*p*EF: heart failure with preserved ejection fraction; HF*r*EF: heart failure with reduced ejection fraction; IPW: inverse probability weighting; RM: ringgit Malaysia

[a] The mean difference between total cost of each cost component estimated by unweighted and IPW method was compared using independent t-test with level of significance of p<0.05.

[b] Post-hoc analysis TukeyHSD was used to compared the mean difference between total cost of each cost component estimated by unweighted and IPW method across different HF phenotypes with level of significance of p<0.05.
