# Supplementary material for: Financial burden of heart failure in Malaysia: A perspective from the public healthcare system
Source: PLoS One. 2023 Jul 5;18(7):e0288035. doi: 10.1371/journal.pone.0288035 (PMC10321615; doi:10.1371/journal.pone.0288035)
Supplement: S3 Table — (PDF) [file pone.0288035.s003.pdf]

Table S3 The heart failure direct cost burden as a percentage of total healthcare expenditure in 2021

| | |
|---|---|
| Estimated total health expenditure in 2021 (RM million) [a] | 72,700 |
| Base-case estimation of HF cost burden (range), (RM million) | 766.3 (50.5 – 1,929.0) |
| Cost burden as a percentage of total healthcare expenditure | 1.05 (0.07 – 2.66) |

[a] The total health expenditure was obtained from CodeBlue. (2021). Malaysia's health spending estimated to hit 5% GDP in 2021. Social Health Analytics Sdn Bhd. Retrieved 02 July 2022 from https://codeblue.galencentre.org/2021/09/21/malaysias-health-spending-estimated-to-hit-5-gdp-in-2021/
