# Supplementary material for: Financial burden of heart failure in Malaysia: A perspective from the public healthcare system
Source: PLoS One. 2023 Jul 5;18(7):e0288035. doi: 10.1371/journal.pone.0288035 (PMC10321615; doi:10.1371/journal.pone.0288035)
Supplement: S4 Table — (PDF) [file pone.0288035.s004.pdf]

Table S4 Summary of censored data

	**Never censored**	**Ever censored**	**Total**
Number of patients	315	14	329
Percentage of patients (%)	95.7	4.3	100
Periods with data available	3780	111	3891
Potential periods of data	3780	168	3948
Periods with data available (%)	100	66.07	98.56
Periods with censored data, (%)	0	33.93	1.44
